# Improved survival in newly diagnosed metastatic prostate cancer: a National Prostate Cancer Audit study

**DOI:** 10.1111/bju.70432

**Published:** 2026-10-08

**Authors:** Arjun Nathan, Matthew G. Parry, Emily Mayne, Adrian Cook, Justin Liu, Raghav Varma, Will Cuningham, Joanna Dodkins, Marina Parry, Julie Nossiter, Ajay Aggarwal, Jan van der Meulen, Alison Tree, Noel Clarke

**Affiliations:** ^1^ Department of Health Services Research & Policy London School of Hygiene & Tropical Medicine London UK; ^2^ National Cancer Audit Collaborating Centre Clinical Effectiveness Unit, Royal College of Surgeons of England London UK; ^3^ Department of Surgery and Interventional Science University College London London UK; ^4^ Guy's Cancer Centre Guy's and St Thomas' NHS Foundation Trust London UK; ^5^ Department of Radiology The Royal Marsden NHS Foundation Trust and The Institute of Cancer Research London UK; ^6^ Department of Urology The Christie NHS Foundation Trust Manchester UK; ^7^ Division of Cancer Sciences The University of Manchester Manchester UK

**Keywords:** metastatic, mortality, population, prostate cancer, survival

The treatment landscape for newly diagnosed metastatic hormone‐sensitive prostate cancer (mHSPC) has evolved over the last decade. Clinical trials have shown that treatment intensification in this setting, comprising chemotherapy and/or androgen‐receptor pathway inhibitors, has shown benefit in delaying tumour progression and improving overall survival [1, 2]. More recently, the addition of localised prostate radiotherapy has also shown significant improvement in overall survival for men with low‐burden metastatic disease, a finding supported by a recent large‐scale meta‐analysis [3].

However, clinical trial evidence does not always translate into real‐world clinical practice as patients may be older and frailer than patients enrolled in trials [4]. Recent national data reported underutilisation but gradually increasing use of systemic anti‐cancer treatment intensification at a population level in England [5]. Therefore, it is still unknown whether these trial‐proven treatments are improving survival outcomes at a population level.

We examined trends in 3‐year mortality for men with newly diagnosed mHSPC in England between 2015 and 2022. Men aged ≥18 years presenting with newly diagnosed mHSPC were identified from the National Cancer Registration Dataset. Death status, date of death, and cause of death were provided by the Civil Registration of Deaths dataset. Prostate cancer‐specific mortality was defined using the International Classification of Diseases and Related Health Problems, Tenth Revision code ‘C61’ as the cause leading to death.

All patients were followed up and censored at 3 years after the date of diagnosis, unless death from any cause occurred. Follow‐up data were available until 1 July 2025 and therefore men diagnosed between 1 January 2015 and 30 June 2022 were included in this study.

All‐cause mortality was estimated using the Kaplan–Meier method. Prostate cancer‐specific mortality was estimated using a competing risks framework, with non‐prostate‐cancer death treated as a competing event [6]. A Fine‐Gray competing risks regression model estimated subdistribution hazard ratios for prostate cancer‐specific mortality, treating death from other causes as a competing event, with 95% confidence intervals (CIs). The model was adjusted for year of diagnosis (2015–2022), age group (18–59, 60–69, 70–79, ≥80), comorbidities (Charlson Comorbidity Index), ethnicity, socioeconomic deprivation (Index of Multiple Deprivation) quintile, prostate‐specific antigen, Gleason score, T‐stage, N‐stage, and metastatic substage (M1a, M1b, M1c, and M1x when the substage was unknown). *P*‐values were two‐sided, and a value <0.05 was considered to indicate a statistically significant difference. Data analysis was undertaken using Stata version 17 (StataCorp LLC, College Station, TX, USA).

A total of 45,339 men were identified with newly diagnosed mHSPC over a 7.5‐year period. Median age at diagnosis was 75 years (interquartile range 69–82), and the distribution of age, ethnicity, and deprivation was stable across year of diagnosis. The proportion of men staged as M1a increased from 3.9% in 2015 to 9.0% in 2022, whereas the unknown substage (M1x) decreased from 43.5% to 36.1%.

Three‐year all‐cause mortality decreased from 55.8% (95% CI 54.6–57.1) in 2015 to 47.1% (95% CI 45.3–48.9) in 2022, an absolute reduction of 8.7% (95% CI 6.5–10.9) (Fig. 1). Improvements were consistent at earlier timepoints, with 1‐year all‐cause mortality falling from 23.3% (95% CI 22.2–24.4) to 17.3% (95% CI 15.9–18.7) and 2‐year mortality from 42.8% (95% CI 41.5–44.1) to 34.6% (95% CI 32.8–36.3).

**Fig. 1:**
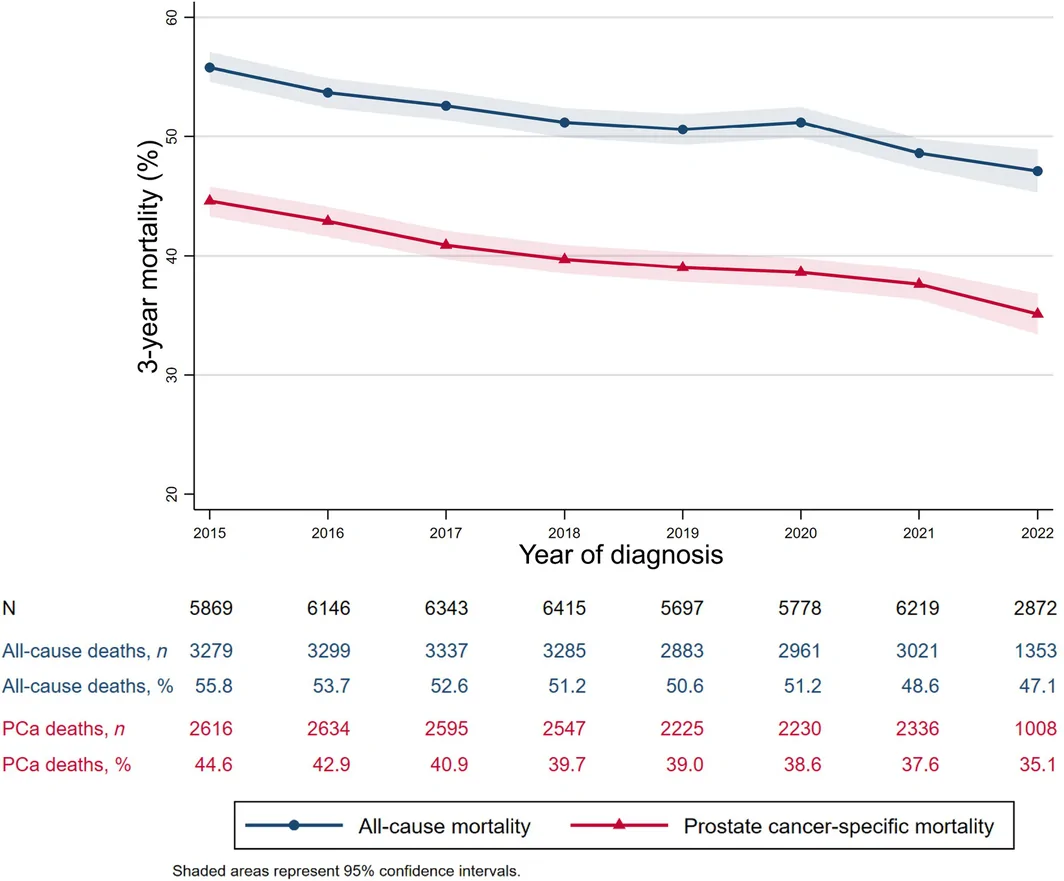
Three‐year all‐cause and prostate cancer‐specific mortality for men newly diagnosed with metastatic prostate cancer (PCa) in England, by year of diagnosis.

Three‐year prostate cancer‐specific mortality decreased from 44.6% (95% CI 43.3–45.8) to 35.1% (95% CI 33.4–36.8), an absolute reduction of 9.5% (95% CI 7.4–11.6). Corresponding reductions at 1 year, from 18.2% (95% CI 17.2–19.2) to 12.5% (95% CI 11.3–13.7), and at 2 years, from 34.4% (95% CI 33.2–35.6) to 25.7% (95% CI 24.1–27.3), were also seen. The absolute reduction in prostate cancer‐specific mortality was similar to the reduction in all‐cause mortality, suggesting that the improvement in all‐cause mortality was driven primarily by fewer prostate cancer deaths.

Year‐on‐year improvements were observed across the study period, although this trend plateaued for men diagnosed in 2020, possibly reflecting the impact of the COVID‐19 pandemic. Accounting for death not caused by prostate cancer and after adjustment for age, comorbidity, ethnicity, socioeconomic deprivation, prostate‐specific antigen, Gleason score, T‐stage, N‐stage, and metastatic substage, a reduction in prostate cancer‐specific mortality was observed with each successive year of diagnosis. The subdistribution hazard ratio for men diagnosed in 2022 was 0.76 (95% CI 0.70–0.81), a 24% lower subdistribution hazard of prostate cancer death than for men diagnosed in 2015 (Table S1).

Our findings represent the final step in a transition from treatment innovation to population‐level impact. We found a near 10% reduction in cancer‐specific 3‐year mortality for men newly diagnosed with mHSPC between 2015 and 2022. This represents a meaningful and measurable survival improvement in real‐world practice following the emergence and increasing utilisation of trial‐proven treatments, as well as possible improvements in staging, earlier diagnosis, and supportive care.

Importantly, the improvement in survival is driven by reductions in prostate cancer mortality, which strengthens the interpretation that improvements in prostate‐cancer‐specific treatment rather than shifts in non‐prostate cancer‐related risk are responsible. Furthermore, the persistence of the improvement after adjustment for case mix makes it unlikely that changes in patient or cancer characteristics are responsible for the improvements in survival.

Population‐level survival remains substantially lower than that in results from clinical trials. We have previously reported a median overall survival of 3 years for men with newly diagnosed metastatic prostate cancer [7]. Our data reflect the older, more comorbid, and less selected patients treated in real‐world practice, and our findings are consistent with other international population‐level studies [8].

Improved imaging may lead to stage migration, whereby men previously classified as non‐metastatic are identified as metastatic, artificially inflating apparent survival (Will Rogers phenomenon). However, the annual number of men diagnosed with metastatic disease remained stable throughout the study period, making stage migration from M0 to M1 an unlikely explanation for the observed improvement.

A key feature of our analysis is that we did not include patient‐level treatment data, as our aim was to capture the net effect of improvements in care at a population level, encompassing the availability and use of novel therapies but also the system‐level circumstances that determine whether patients access them, such as improved staging, timely referral, and appropriate treatment initiation.

In conclusion, survival after a new diagnosis of mHSPC in England improved significantly between 2015 and 2022. The improvement is primarily driven by reductions in prostate cancer death. The findings represent clear evidence of a major step forward in improving the care of men with mHSPC, and continued monitoring of survival and treatment patterns will be essential as treatment pathways evolve further.

## Author Contributions

Conception and design: AN, JVDM, and NC. Acquisition of data: AN, AC, and EM. Analysis and interpretation of data: all authors. Drafting of the manuscript: AN, MGP, JVDM, AT, and NC. Review of final manuscript: all authors.

## Disclosure of Interests

None declared.

## Funding

Arjun Nathan was funded by a National Institute for Health and Care Research (NIHR) Doctoral Research Fellowship (grant ref: NIHR304655) and has received funding from The Urology Foundation and the Charles Reynolds Foundation. Alison Tree is supported by the Cancer Research UK Radiation Research Centre of Excellence at The Institute of Cancer Research and The Royal Marsden NHS Foundation Trust (grant ref: A28724 and RRCOER‐Jun24/100006) and by a Cancer Research UK Programme grant (ref: C33589/A28284). Alison also acknowledges NHS funding to the NIHR Biomedical Research Centre at The Royal Marsden and The Institute of Cancer Research. The views expressed in this publication are those of the author(s) and not necessarily those of the NHS, the NIHR or the Department of Health and Social Care. This study was undertaken alongside the National Prostate Cancer Audit. The audit is commissioned by the Healthcare Quality Improvement Partnership (HQIP) as part of the National Clinical Audit and Patient Outcomes Programme, funded by NHS England and the Welsh Government (https://www.hqip.org.uk/national‐programmes). Neither HQIP nor NHS England had any involvement in the study design; in the collection, analysis, and interpretation of data; in the writing of the report; or in the decision to submit the article for publication.

## Ethical Approval

This study was conducted as part of the National Prostate Cancer Audit (NPCA), which has established regulatory approval, data security, and governance procedures. Since this research involves anonymised secondary data, UK National Research Ethics Committee approval has not been sought, per their guidelines. The study was performed per the Declaration of Helsinki.

## Supporting information


**Table S1.** Fine‐Gray competing risk regression model of associations between year of diagnosis (2015–2022) and prostate cancer (PCa)‐specific mortality, adjusted for patient and tumour characteristics.

## Data Availability

Data are available on reasonable request. Data are available from the Data Access Request Service, NHS England (https://digital.nhs.uk/services/data‐access‐request‐service‐dars). Arjun Nathan had full access to all the data in the study and takes responsibility for the integrity of the data and the accuracy of the data analysis. Statistical coding data are available upon reasonable request.
