# Supplementary material for: Improved survival in newly diagnosed metastatic prostate cancer: a National Prostate Cancer Audit study
Source: BJU Int. 2026 Oct 8;138(6):1007–10. doi: 10.1111/bju.70432 (PMC13741100; doi:10.1111/bju.70432)
Supplement: Supplementary file 1 [file BJU-138-1007-s001.docx]

**Supplementary Material**

**Table 1. Fine-Gray competing risk regression model of associations between year of diagnosis (2015-2022) and prostate cancer (PCa) -specific mortality, adjusted for patient and tumour characteristics**

|  | **Study Population** | | **PCa Mortality** | | | | |
| --- | --- | --- | --- | --- | --- | --- | --- |
|  | **n** | **%** | **n** | **%** | **aSHR** | **95% CI** | **p value** |
| **Year of diagnosis** |  |  |  |  |  |  |  |
| 2015 (reference) | 5,869 | *12.9* | 2,616 | *44.6* | 1 | — | *—* |
| 2016 | 6,146 | *13.6* | 2,634 | *42.9* | 0.96 | 0.91–1.01 | *0.149* |
| 2017 | 6,343 | *14.0* | 2,595 | *40.9* | 0.89 | 0.85–0.94 | *<0.001* |
| 2018 | 6,415 | *14.2* | 2,547 | *39.7* | 0.88 | 0.83–0.93 | *<0.001* |
| 2019 | 5,697 | *12.6* | 2,225 | *39.1* | 0.87 | 0.82–0.92 | *<0.001* |
| 2020 | 5,778 | *12.7* | 2,230 | *38.6* | 0.80 | 0.75–0.84 | *<0.001* |
| 2021 | 6,219 | *13.7* | 2,336 | *37.6* | 0.80 | 0.75–0.85 | *<0.001* |
| 2022* | 2,872 | *6.3* | 1,008 | *35.1* | 0.75 | 0.70–0.81 | *<0.001* |
| **Age group (years)** |  |  |  |  |  |  |  |
| 18–59 (reference) | 2,817 | *6.2* | 948 | *33.7* | 1 | — | *—* |
| 60–69 | 9,649 | *21.3* | 3,089 | *32.0* | 0.90 | 0.84–0.97 | *0.004* |
| 70–79 | 17,274 | *38.1* | 6,030 | *34.9* | 0.96 | 0.90–1.03 | *0.290* |
| 80+ | 15,599 | *34.4* | 8,124 | *52.1* | 1.33 | 1.24–1.43 | *<0.001* |
| **Charlson Comorbidity Index** |  |  |  |  |  |  |  |
| 0 (reference) | 35,565 | *78.4* | 13,600 | *38.2* | 1 | — | *—* |
| 1 | 4,370 | *9.6* | 1,923 | *44.0* | 1.11 | 1.06–1.17 | *<0.001* |
| 2+ | 5,404 | *11.9* | 2,668 | *49.4* | 1.22 | 1.16–1.27 | *<0.001* |
| **Ethnicity** |  |  |  |  |  |  |  |
| White (reference) | 41,281 | *91.0* | 16,651 | *40.3* | 1 | — | *—* |
| Black | 1,200 | *2.6* | 448 | *37.3* | 0.87 | 0.79–0.96 | *0.004* |
| Asian | 705 | *1.6* | 285 | *40.4* | 0.98 | 0.87–1.10 | *0.682* |
| Mixed | 189 | *0.4* | 70 | *37.0* | 0.91 | 0.72–1.15 | *0.445* |
| Other | 453 | *1.0* | 169 | *37.3* | 0.95 | 0.82–1.11 | *0.506* |
| Missing | 1,511 | *3.3* | 530 | *37.7* | 1.01 | 0.92–1.11 | *0.796* |
| **Index of Multiple Deprivation** |  |  |  |  |  |  |  |
| 1 – Least deprived (reference) | 10,201 | *22.5* | 3,914 | *38.4* | 1 | — | *—* |
| 2 | 10,294 | *22.7* | 3,929 | *38.2* | 0.99 | 0.95–1.04 | *0.826* |
| 3 | 9,565 | *21.1* | 3,833 | *40.1* | 1.07 | 1.02–1.12 | *0.004* |
| 4 | 8,090 | *17.8* | 3,378 | *41.8* | 1.13 | 1.08–1.18 | *<0.001* |
| 5 – Most deprived | 7,189 | *15.9* | 3,137 | *43.6* | 1.21 | 1.15–1.27 | *<0.001* |
| **PSA at diagnosis (ng/mL)** |  |  |  |  |  |  |  |
| 0–149 (reference) | 14,808 | *32.7* | 4,728 | *31.9* | 1 | — | *—* |
| 150+ | 12,787 | *28.2* | 5,528 | *43.2* | 1.21 | 1.16–1.26 | *<0.001* |
| Missing | 17,744 | *39.1* | 7,935 | *44.7* | 1.26 | 1.22–1.31 | *<0.001* |
| **Gleason score** |  |  |  |  |  |  |  |
| 6-7 (reference) | 4,041 | *8.9* | 656 | *16.2* | 1 | — | *—* |
| 8-10 | 18,034 | *39.8* | 5,795 | *32.1* | 1.99 | 1.84–2.16 | *<0.001* |
| Missing | 23,264 | *51.3* | 11,740 | *50.5* | 2.96 | 2.73–3.21 | *<0.001* |
| **T stage** |  |  |  |  |  |  |  |
| 1-2 (reference) | 3,391 | *7.5* | 989 | *29.2* | 1 | — | *—* |
| 3-4 | 24,192 | *53.4* | 8,568 | *35.4* | 1.15 | 1.08–1.23 | *<0.001* |
| Missing | 17,756 | *39.2* | 8,634 | *48.6* | 1.26 | 1.18–1.36 | *<0.001* |
| **N stage** |  |  |  |  |  |  |  |
| 0 (reference) | 10,087 | *22.3* | 3,080 | *30.5* | 1 | — | *—* |
| 1 | 16,484 | *36.4* | 6,117 | *37.1* | 1.27 | 1.22–1.33 | *<0.001* |
| Missing | 18,768 | *41.4* | 8,994 | *47.9* | 1.23 | 1.17–1.29 | *<0.001* |
| **Metastatic substage** |  |  |  |  |  |  |  |
| 1a | 2,591 | *5.7* | 661 | *25.5* | 0.65 | 0.60–0.70 | *<0.001* |
| 1b (reference) | 25,487 | *56.2* | 10,459 | *41.0* | 1 | — | *—* |
| 1c | 2,203 | *4.9* | 1,194 | *54.2* | 1.48 | 1.39–1.58 | *<0.001* |
| 1x | 15,058 | *33.2* | 5,877 | *39.0* | 0.97 | 0.94–1.00 | *0.043* |

aSHR = adjusted Subdistribution Hazard Ratio, *year of diagnosis 2022 includes men diagnosed between January and July only
